# Immune Response to SARS-CoV-2 Infections in Children with Secondary Immunodeficiencies

**DOI:** 10.1007/s10875-022-01365-8

**Published:** 2022-09-23

**Authors:** Karolina Kuczborska, Ewelina Krzemińska, Piotr Buda, Edyta Heropolitańska-Pliszka, Barbara Piątosa, Janusz Książyk

**Affiliations:** 1 Department of Pediatrics, Nutrition and Metabolic Disorders, Children’s Memorial Health Institute, Av. Dzieci Polskich 20, 04-730 Warsaw, Poland; 2 Department of Immunology, Children’s Memorial Health Institute, Av. Dzieci Polskich 20, 04-730 Warsaw, Poland; 3 Histocompatibility Laboratory, Children’s Memorial Health Institute, Av. Dzieci Polskich 20, 04-730 Warsaw, Poland

**Keywords:** SARS-CoV-2, COVID-19, Cancer, Kidney transplantation, Immunodeficiency, Children, Serology, Lymphocyte subsets

## Abstract

**Background and Purpose:**

It is a matter of research, whether children with immunodeficiencies are able to generate an effective immune response to prevent SARS-CoV-2 reinfection. This study aimed to evaluate and compare the seroconversion rates and changes of lymphocyte subsets during COVID-19 in immunocompetent children and those with secondary immunodeficiencies.

**Methods:**

In 55 children — 28 immunocompromised and 27 immunocompetent — hospitalized with confirmed SARS-CoV-2 infection, the level of IgG antibodies against the Spike protein was determined on two to three occasions. In those children from the study group whose immunosuppressive treatment did not alter during the study (*n* = 13) and in selected children from the control group (*n* = 11), flow cytometric evaluation of lymphocyte subsets was performed twice — 2 weeks and 3 months post-infection**.**

**Results:**

Seroconversion reached 96.3% in both studied groups; however, the immunocompromised cohort achieved lower titers of detectable anti-S antibodies. There was no correlation between seroconversion or titers of antibodies and the total number of lymphocytes or their subsets. In the immunocompetent cohort, we reported a significant decrease in NK cells during the infection. In this group and the entire study population, a positive correlation was noticed between the CD4 + /CD8 + T cell ratio and the severity of COVID-19 pneumonia.

**Conclusions:**

Children with secondary immunodeficiencies seroconvert in equal percentages but with a significantly lower titer of anti-S antibodies compared to their immunocompetent peers. The lower number of NK cells in the immunocompetent cohort may result from their participation in antiviral immunity, whereas reduced CD4 + /CD8 + T cell ratios among immunocompromised children may be a protective factor against a severe COVID-19.

## Introduction

Since the onset of the coronavirus disease 2019 (COVID-19) pandemic caused by severe acute respiratory syndrome coronavirus 2 (SARS-CoV-2), scientists worldwide have been investigating the induction of protective immunity following previous infections and/or successful vaccination as the only way to suppress transmission. Despite two and a half years since the pandemic started, our knowledge in this area remains insufficient, especially in immunocompromised pediatric patients. Despite initial concerns about the course of SARS-CoV-2 infections in this group, we are now aware that they usually exhibit mild or asymptomatic diseases [1, 2].

Nevertheless, despite mild symptoms and rare complications, COVID-19 continues to be a significant clinical problem in this group. It has been proven that SARS-CoV-2 infections frequently cause interruptions in oncological treatment, which may have implications for the success of treatment [3]. Additionally, in kidney transplant children, it may lead to proteinuria and acute kidney injury (AKI), which may affect the long-term survival of the graft [4]. Therefore, it is important to protect immunocompromised children against COVID-19. To achieve this, it is crucial to understand the immunopathogenesis of the infection and the immune response against it. While humoral and to a lesser extent cellular immunity against the virus is well described in immunocompetent convalescent children, it is still a matter of research, whether immunocompromised children are able to generate an effective immune response to reinfection.

In this regard, this study aimed to evaluate and compare seroconversion rates following COVID-19, as well as changes of lymphocyte subsets during SARS-CoV-2 infections between immunocompetent children and those with secondary immunodeficiencies, who were admitted to the COVID-19 subunit of the tertiary referral hospital in Warsaw, Poland.

## Materials and Methods

### Study Group

A group of 55 children (22 girls and 33 boys) hospitalized in the COVID-19 Subunit at the Department of Pediatrics, Nutrition, and Metabolic Disorders, Children’s Memorial Health Institute in Warsaw, Poland, between October 1, 2021, and March 15, 2022, participated in this prospective study.

### Inclusion Criteria

1. Age: 3 months to 18 years
2. SARS-CoV-2 infection confirmed by nasopharyngeal swab, followed by reverse transcription polymerase chain reaction (RT-PCR) or rapid antigen test performed before or during hospitalization

### Exclusion Criteria

1. Vaccination against SARS-CoV-2
2. Previous diagnosis of COVID-19

### Division of the Group

Study participants were stratified into two separate cohorts, depending on whether they had secondary immunodeficiencies. Children with immunodeficiencies (*n* = 28) comprised 50.9% of the study participants, while the control group (*n* = 27) comprised 49.1%. Inclusion in the immunodeficient cohort was dependent on the presence of a tumor undergoing oncological treatment or kidney transplantation.

### SARS-CoV-2 S1 IgG Assay

In all children included in this study, the level of IgG antibodies against the receptor-binding domain (RBD) of the S1 subunit of the spike (S) protein of SARS-CoV-2 was determined on two or three occasions. For this purpose, the AdviseDx SARS-CoV-2 IgG II assay (Abbott Laboratories, Abbott Park, IL) was used and performed according to the manufacturer’s instructions. Results of the AdviseDx SARS-CoV-2 IgG II assay are reported in arbitrary units (AU)/mL, with the cutoff for seroconversion established at 50.0 AU/mL [5]. The first analysis was performed on samples from all children at an interval of 2 weeks after obtaining a positive RT-PCR or rapid antigen test result. In seronegative children, the analysis was repeated 3 weeks post-infection. In addition, within 3 months post-infection, all study participants had another test to assess the persistence of antibody levels.

### Flow Cytometry

In children from the study group whose immunosuppressive treatment did not alter during the study (*n* = 13) and in selected children from the control group (*n* = 11), flow cytometric evaluation of lymphocyte subsets was performed twice — 2 weeks and 3 months after obtaining a positive RT-PCR or rapid antigen test result. The assessed lymphocyte subset panel included T cells (CD3 + /CD45 +), cytotoxic T cells (CD3 + CD8 + /CD45 +), helper T cells (CD3 + CD4 + /CD45 +), NK cells (CD16 + CD56 + CD3 − /CD45 +), and B cells (CD19 + /CD45 +). Their distribution and absolute cell counts were determined using the lyse-no-wash approach and Multitest six-color cocktails of antibodies and Trucount tubes (Becton Dickinson, cat. no. 644611). The staining procedure was performed according to the manufacturer’s instructions. Initially, 50-μL blood samples from patients were incubated with optimally titered antibodies for 15 min at room temperature. After the incubation period, erythrocyte lysis was performed using 0.45 mL of BD FACSLysing Solution (Becton Dickinson, cat. no. 349202) diluted according to the manufacturer’s instructions. Identification of lymphocyte subsets, i.e., T lymphocytes (including CD4 and CD8 cells), B lymphocytes, and NK cells, was performed according to standard procedures [6]. The absolute number of individual subsets was calculated based on the proportion of the respective cell subpopulation and absolute lymphocyte count.

### *COVID-19 Severity — Definitions *[7]

• Asymptomatic — children with no symptoms of COVID-19
• Mild — children with various signs and symptoms that generally do not require hospitalization (e.g., moderate fever, cough, sore throat, malaise, headache, muscle pain, nausea, vomiting, diarrhea, and loss of taste and smell), but who do not have shortness of breath, dyspnea, or abnormal chest imaging
• Moderate — children who show evidence of lower respiratory disease during clinical assessment or imaging with an oxygen saturation (SpO2) ≥ 94% on room air at sea level
• Severe — children with SpO2 < 94% on room air at sea level, tachypnea, or lung infiltrates > 50%

### Statistical Analysis

All data were analyzed using Microsoft Excel and Statistica 13 software. Quantitative data was evaluated using the Mann–Whitney *U* test or Spearman’s rank correlation coefficient when the distribution was significantly different from a Gaussian distribution. Normality was determined using the Shapiro–Wilk test. Qualitative data was compared using the chi-square test. The probability value of *p* < 0.05 was considered to be statistically significant.

### Ethical Considerations

The study was conducted in accordance with the Declaration of Helsinki and approved by the institutional Ethics Committee of the Children’s Memorial Health Institute in Warsaw, Poland (No. 41/KBE/2021). Parents and children were informed about the aim and methods of the study. Parents and patients over 12 years of age provided their written consent to participate in the study.

## Results

### Characteristics of the Group

Immunocompromised and immunocompetent children did not differ significantly in terms of age and sex. However, they did differ in terms of COVID-19 severity, which was much more often symptomatic and severe in the immunocompetent cohort. Within the immunodeficient cohort, the most common immunosuppressive factor was tumor undergoing oncological treatment and a less numerous group was composed of kidney transplant recipients. Detailed data on the characteristics of both cohorts are presented in Table 1.

**Table 1:** Characteristics of the participants

	ID ( +) ***n*** = 28	ID ( −) ***n*** = 27	***p***
Age, y, median (range)	6 (3.8–10.3)	3 (0.9–11.5)	
< 1 year old	2 (7.1%)	8 (29.6%)	0.20
1–5 years old	11 (39.3%)	9 (33.3%)	
6–10 years old	8 (25.8%)	2 (7.4%)	
> 10 years old	7 (22.6%)	8 (29.6%)	
Sex			
Female	12 (42.9%)	10 (37.0%)	0.66
Male	16 (57.1%)	17 (63.0%)	
Severity of COVID-19			
Asymptomatic	8 (28.6%)	5 (18.5%)	< *0.01*
Mild	16 (57.1%)	6 (22.2%)	
Moderate	2 (7.1%)	10 (37.0%)	
Severe	2 (7.1%)	6 (22.2%)	
Pneumonia	4 (14.3%)	16 (59.3%)	< *0.01*
Immunodeficiency			
Oncological treatment	23 (82.1%)	N/A	N/A
RTH	6 (26.1%)		
VCR	3 (13.0%)		
MTH	1 (4.3%)		
VCR, CDDP	1 (4.3%)		
VP, DTIC	1 (4.3%)		
VCR, ADM, IF	1 (4.3%)		
VCR, CDDP, CCNU	1 (4.3%)		
VCR, VP, CBP	1 (4.3%)		
VCR, IF, ACT-D	1 (4.3%)		
VCR, ADM, CTX	1 (4.3%)		
VCR, CTX, prednisone	1 (4.3%)		
VP, CDDP, CB, PAL	1 (4.3%)		
VCR, VP, ADM, IF, PC	1 (4.3%)		
VCR, MTX, VP, CTX, CDDP	2 (8.7%)		
VBL, CDDP, CTX, BLM, ADM, VP	1 (4.3%)		
Kidney transplantation	5 (17.9%)		
Prednisone, tacrolimus, mycophenolate mofetil	5 (100%)		

*ACT-D*, actinomycin D; *ADM*, doxorubicin; *BLM*, bleomycin; *CB*, chlorambucil; *CBP*, carboplatin; *CCNU*, lomustine; *CDDP*, cisplatin; *COVID-19*, coronavirus disease 2019; *CTX*, cyclophosphamide; *DTIC*, dacarbazine; *ID (* +*)*, children with secondary immunodeficiency; *ID ( −)*, children without secondary immunodeficiency; *IF*, ifosfamide; *PAL*, palbociclib; *PC*, paclitaxel; *RTH*, radiotherapy; *VCR*, vincristine; *VP*, etoposide

During the observation period, reinfection with another variant of the virus was confirmed in one immunocompetent (3.7%) and two immunocompromised patients (7.1%). All of them had detectable IgG antibodies against the SARS-CoV-2 S protein at the time. The course of reinfection in the immunocompetent boy was mild, as was the first episode of his COVID-19, but between infections, the patient underwent severe multisystem inflammatory syndrome in children (MIS-C) complicated by pulmonary embolism. In turn, two oncological girls were reinfected only with low symptoms (1 day of fever), while in the first COVID-19 they developed moderate pneumonia.

### Seroconversion

Seroconversion rates 2 weeks post-infection were lower among immunocompromised children than in immunocompetent children, but the difference did not reach statistical significance (*p* = 0.17; OR = 2.26; 95% CI = 0.69, 7.39). Moreover, these percentages were comparable at 4 weeks and 3 months post-infection. It should be noted that children with immunodeficiency both at initial and post-infection periods produced significantly lower antibody titers (Table 2).

**Table 2:** Seroconversion and anti-S IgG titer in 2 weeks, 3 weeks, and 3 months post-infection in children with and without immunodeficiency. *p* values were calculated using the Mann–Whitney *U* test for quantitative data (anti-S titer), whereas qualitative data (seroconversion) were compared using the chi-square test

		ID ( +) ***n*** = 28	ID ( −) ***n*** = 27	***p***
2 weeks post-infection	Seroconversion	17 (60.7%)	21 (77.8%)	0.17
	Anti-S IgG titer — median (range) [AU/mL]	171.5 (27.9–874.7)	967.2 (178.0–3168.4)	*0.03*
3 weeks post-infection	Seroconversion	27 (96.4%)	26 (96.3%)	0.98
3 months post-infection	Seroconversion	26 (92.9%)	26 (96.3%)	0.57
	Anti-S IgG titer — median (range) [AU/mL]	236.6 (140.5–1109.1)	750.1 (384.9–1956.15)	*0.04*

*ID (* +*)*, children with secondary immunodeficiency; *ID ( −)*, children without secondary immunodeficiency

### Lymphocyte Subsets

In the immunocompetent cohort, lymphocyte counts during COVID-19 remained within normal ranges. As for the analyzed subsets, we only noticed a significant decrease in NK cells during the infection. All other differences did not reach statistical significance. Among immunocompromised children, the number of all subpopulations was reduced due to the treatment of the underlying disease; however, we did not notice any significant differences in the number of individual subsets during infection and 3 months after its diagnosis (Table 3).

**Table 3:** Lymphocyte subsets 2 weeks and 3 months post-infection in children with and without immunodeficiency-absolute values-median (range). *p* values were calculated using the Mann–Whitney *U* test to compare the number of each lymphocyte subset 2 weeks and 3 months after the confirmation of SARS-CoV-2 infection

Variables	ID ( +)			ID ( −)		
	2 weeks post-infection	3 months post-infection	*p*	2 weeks post-infection	3 months post-infection	*p*
Lymphocytes [n/µL]	833 (338–1580)	590 (422–2076)	0.92	4099 (3110–6042)	3339 (2793–5510)	0.85
T cells [n/µL]	539 (269–1257)	331 (284–1546)	0.70	2749 (1732–4020)	2584 (1940–3582)	0.85
CD4 + T cells [n/µL]	265 (94–705)	129 (103–635)	0.08	2416 (1196–3242)	1791 (944–2324)	0.22
CD8 + T cells [n/µL]	269 (137–468)	223 (94–532)	0.14	667 (514–1012)	855 (634–1113)	0.48
CD4 + /CD8 + ratio	1.2 (0.6–1.4)	0.8 (0.7–1.4)	0.97	3.1 (2.4–3.9)	2.5 (1.4–3.6)	0.35
B cells [n/µL]	35 (25–155)	78 (49–267)	0.37	1192 (1087–1606)	1073 (463–1347)	0.44
NK cells [n/µL]	73 (45–98)	82 (69–235)	0.37	225 (195–276)	360 (294–558)	*0.02*

*ID (* +*)*, children with secondary immunodeficiency; *ID ( −)*, children without secondary immunodeficiency

We also did not observe any significant correlations between individual lymphocyte subsets or the CD4 + /CD8 + ratios and the seroconversion rate or achieved antibody titers — in the whole study group and the subgroups of children with and without immunodeficiency (*p* > 0.05).

### Course of COVID-19 and Immunological Response

**Fig. 1:**
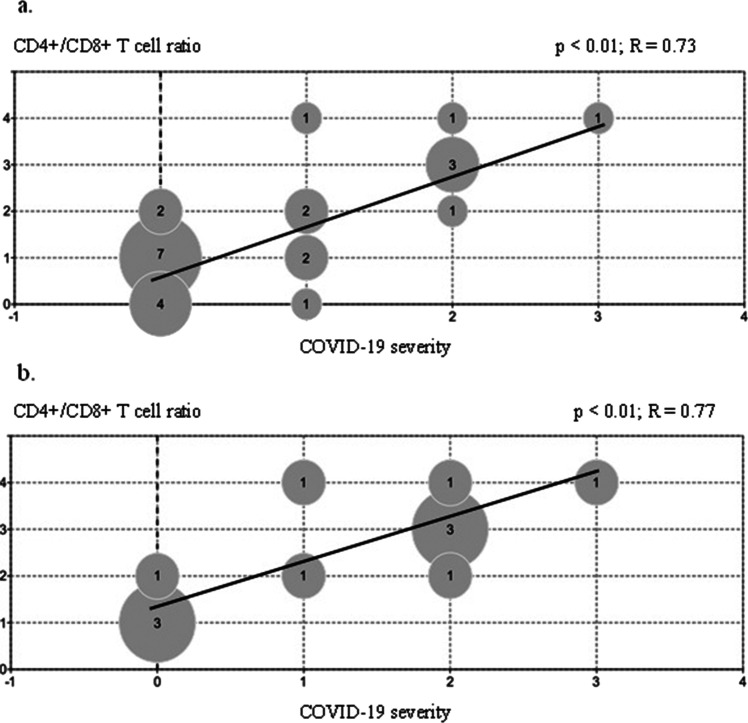
Spearman’s rank correlation between CD4 + /CD8 + T cell ratios and COVID-19 severity among **a** the entire study population and **b** the immunocompetent cohort. The numbers in the circles represent the number of patients. The degrees of COVID-19 severity are described in the “Materials and Methods” section, with 0 as an asymptomatic course, 1 — mild, 2 — moderate, and 3 — severe

In the entire study population and the immunocompetent cohort, we noticed a positive correlation between CD4 + /CD8 + T cell ratios and the severity of SARS-CoV-2 infection — the higher the ratio, the greater the risk of developing and the greater the severity of COVID-19 pneumonia (*p* < 0.01; *R* = 0.73; *p* < 0.01; *R* = 0.77, respectively) (Fig. 1). No other predictors of the severe course of COVID-19 were found in the immunocompetent cohort, and since only mild and asymptomatic courses were observed in this subgroup of immunocompromised children, these predictors could not be assessed (Table 4). Nevertheless, we did not observe any correlations between the severity of disease and seroconversion rates as well as the titers of produced antibodies (*p* > 0.05).

**Table 4:** Lymphocyte subsets 2 weeks post-infection in children with and without immunodeficiency categorized according to the severity of COVID-19-absolute values-median (range). *p* values were calculated using the Mann–Whitney *U* test to compare the number of each lymphocyte subset

Variables	ID ( +)			ID ( −)		
	Asymptomatic/mild	Moderate/severe	*p*	Asymptomatic/mild	Moderate/severe	*p*
Lymphocytes [n/µL]	833 (338–1580)	N/A	N/A	4373 (3130–6493)	4683 (3198–6610)	1.0
T cells [n/µL]	539 (269–1257)			2990 (1988–4488)	3006 (1988–4245)	0.93
CD4 + T cells [n/µL]	265 (94–705)			1965 (1217–2939)	2416 (1435–3242)	0.35
CD8 + T cells [n/µL]	269 (137–468)			973 (691–1489)	582 (423–960)	0.32
CD4 + /CD8 + ratio	1.2 (0.6–1.4)			1.9 (1.7–2.1)	3.5 (3.1–4.1)	< 0.01
B cells [n/µL]	35 (25–155)			1166 (818–1582)	1200 (1169–1805)	0.79
NK cells [n/µL]	73 (45–98)			254 (200–529)	218 (202–323)	0.79

*ID (* +*)*, children with secondary immunodeficiency; *ID (* −*)*, children without secondary immunodeficiency

## Discussion

Although the presence of neutralizing antibodies against SARS-CoV-2 proteins does not fully protect against infection, it significantly reduces its risk. Letizia et al. proved that seropositive convalescent young adults had as much as five times lower risk of reinfection compared to a seronegative comparison group [8]. Hence, although seroconversion is not a guarantee of effective neutralization activity, it does indicate the acquisition of at least partial immunity [9]. Moreover, the early adaptive humoral immune response involving seroconversion has a proven effect on slowing down the replication of the virus during infections and its faster elimination [10]. In addition, slow elimination and persistence of the virus can lead to complications such as mutations in viral proteins and long-COVID symptoms [11]. Immunocompromised children still have low vaccination rates. Additionally, they are exposed to significant clinical implications of SARS-CoV-2 infections, such as delay and modification in the treatment of their underlying disease [3], deterioration of graft function [4], and a tendency for virus persistence predisposing to the development of long-COVID symptoms, as well as reactivation of latent herpesviruses [11]. Therefore, the question of whether this group can develop an effective immune response to reinfections and how long it may persist is incredibly important.

In research studies regarding the presence of antibodies against SARS-CoV-2 among immunocompromised children, the authors have focused on the seroprevalence in specific populations rather than on the seroconversion of children with confirmed SARS-CoV-2 infections. Therefore, we aimed to explore the latter issue. Mayanskiy et al. revealed a seroconversion of 92% in 18 pediatric oncology patients by 3 weeks post-infection and in 100% by 6 weeks post-infection, which is similar to our results. By 18 weeks after the onset of COVID-19, this seropositivity rate declined to 54%. However, the authors did not compare the level of antibodies with an immunocompetent control group [12]. On the other hand, researchers from Hungary assessed the level of antibodies in 10 children with cancer at an interval of 1 to 4.5 months after the onset of COVID-19. They detected a seroconversion level of only 60% [13].

As for pediatric solid organ transplant (SOT) recipients, Talgam-Horshi et al. revealed a 96% seroconversion rate among 25 patients. However, they did not present the antibody levels or compare them with a control group [14]. Meanwhile, researchers from Saudi Arabia observed eight pediatric kidney transplant (PKT) recipients and showed their ability to maintain detectable levels of antibodies at a median follow-up time of 75 days [15]. We, in turn, observed these patients to have a detectable antibody titer for at least 90 days. None of the above-mentioned authors reported the number of patients reinfected with SARS-CoV-2 during their observation, despite having detectable titers of antibodies against the S protein. In our study, the reinfection rate was 5.5% (*n* = 3; one immunocompetent and two immunocompromised patients).

Various researchers have also assessed lymphocyte subsets in immunocompetent children with COVID-19. They proved that children, unlike adults, rarely have lymphopenia in the course of SARS-CoV-2 infections, and if present, its degree correlates with the severity of the disease and usually affects all lymphocyte subsets [16]. Our study did not reveal a tendency for lymphopenia in children, regardless of the severity of COVID-19. However, as for the values of individual lymphocyte subsets, the scientific data is not unequivocal. According to Mahmoudi et al., children with SARS-CoV-2-induced pneumonia have higher CD8 + T cell counts and lower CD4 + /CD8 + T cell ratios [15]. On the one hand, CD8 + T cells are responsible for destroying virus-infected cells and thus limiting their replication and spread [17]. On the other hand, they can cause damage to lung tissue if they are not sufficiently regulated [18]. However, researchers from China present differing results, according to which the more severe the course of COVID-19 and greater lung involvement, the higher the CD4 + /CD8 + T cell ratio [19]. This is in line with our results and could at least partially explain the milder course of SARS-CoV-2 infections, usually asymptomatic or mild without lung involvement, in immunocompromised children with significantly lower CD4 + /CD8 + ratios. It should be noted, however, that our study population differed from the groups mentioned above, which may substantially impact the differences in obtained results.

One of the most important features we observed in the lymphocyte subsets of immunocompetent children was the reduction of NK cells during COVID-19, which may be due to their participation in the defense against SARS-CoV-2. None of the above-mentioned studies in children has addressed this issue. However, it has been studied in more detail among adults. NK cells are part of innate immunity and play a key role in tackling viral infections. They eliminate virus-infected cells either directly through the degranulation of cytotoxic granules or through the secretion of cytokines and chemokines that modulate the activity of other immune cells [20]. Many researchers have demonstrated a reduced number of NK cells in patients with COVID-19 [21–23]. Krämer et al. proved that the activity of NK cells producing IFN-γ and TNF-α in response to SARS-CoV-2 infection leads to a reduction of viral proteins [21]. Unfortunately, these cells have also been shown to be dysfunctional in severe COVID-19, which reduces their participation in antiviral immunity [21, 24]. To the best of our knowledge, this phenomenon has not been analyzed in children, who are often characterized by different immune mechanisms. Thus, we are the first to report a reduced number of NK cells in the immunocompetent pediatric group during COVID-19, but unfortunately, we did not examine the activity of these cells. Therefore, this issue requires further research.

Our study has some limitations. The first one is a small size of the study group, especially the subgroup in which lymphocyte subsets were assessed. The second one is the disparity between the course of SARS-CoV-2 infection in immunocompetent and immunocompromised children, which can make it difficult to deduct conclusions on the protective effect of a lower CD4/CD8 ratio. However, due to the generally milder course of COVID-19 in the immunocompromised group of children, this limitation is difficult to omit. Another limitation is the lack of evaluation of a specific T-cell response to the infection in both groups of children, which would significantly complement the information about the humoral response. Although the specific T-cell response is the most important element of antiviral immunity, yet impossible to perform in our Institute at the beginning of our study, we decided to base on information about the humoral response, especially since antibody titers have been proven to correlate with the number of specific T-lymphocytes [25, 26]. However, further studies on a larger group with a similar course of COVID-19, including not only humoral, but also a cellular response to the infection, are necessary to reliably determine the immune response to SARS-CoV-2 infection in children with secondary immunodeficiencies.

## Conclusions

After contracting COVID-19, children with secondary immunodeficiencies seroconvert with the production of anti-S antibodies in an equal percentage to their immunocompetent peers. However, this seroconversion tends to occur later and leads to the production of significantly lower titers of detectable antibodies compared to the control group. Nevertheless, both seroconversion and titers of these antibodies do not depend on the number of total lymphocytes or their subsets.

In the immunocompetent cohort, the number of NK cells decreased during SARS-CoV-2 infections, which can be explained by their recruitment into affected tissues and participation in antiviral immunity. However, no significant differences in lymphocyte subsets during COVID-19 were observed in children with secondary immunodeficiencies.

Based on our study group, there seems to be a correlation between the CD4 + /CD8 + T cell ratio and the severity of SARS-CoV-2 infections — the higher the ratio, the greater the risk of developing and severity of COVID-19 pneumonia. Hence, a reduced ratio in immunocompromised children may be a protective factor against a severe course of COVID-19. However, due to the disparate severity of the disease course in both groups, this hypothesis requires confirmation in larger studies.

## Data Availability

Not applicable.

## Data Availability

The datasets analyzed during the current study are available from the corresponding author on reasonable request.
